# B cell-derived exosomal miR-34a-5p mediates radiation-induced bystander effect through ferroptosis

**DOI:** 10.1515/med-2026-1375

**Published:** 2026-03-20

**Authors:** Wenshan Zhou, Jie Yu, Kui Ma, Fang Wang, Jun Deng, Gangtao Sun

**Affiliations:** Department of Radiation Hygiene Monitoring and Evaluation, Hubei Provincial Center for Disease Control and Prevention, Wuhan, Hubei, China; Key Laboratory of Radiological Protection and Nuclear Emergency, China CDC, National Institute for Radiological Protection, Chinese Center for Disease Control and Prevention, Beijing, China

**Keywords:** exosome, B cell, miR-34a-5p, radiation-induced bystander effect, ferroptosis

## Abstract

**Objectives:**

The radiation-induced bystander effect (RIBE) is a destructive reaction that occurs in non-irradiated cells. Exosomes, as an important intercellular information carrier, are considered potential mediators of RIBE, but their role in B cells remains unclear.

**Methods:**

B cell line IM-9 cells were irradiated to obtain exosomes for small RNA sequencing, and cell assays were used to assess the key miRNA’s role in non-irradiated B cell ferroptosis.

**Results:**

Exosomes isolated from irradiated and non-irradiated B cells were well characterized, displaying typical cup-shaped morphology (50–150 nm) and expressing exosomal markers ALIX and TSG101. miR-34a-5p was identified to be a key miRNA in regulating ferroptosis, and significantly upregulated in irradiated B cell derived exosomes (IR-exo). IR-exo remarkedly promoted ferroptosis of non-irradiated IM-9 cells, as evident by enhanced lactate dehydrogenase activity and lipid peroxidation, and reduced SLC7A11, GPX4 and FTH1 expression. However, miR-34a-5p silencing in IR-exo reversed IR-exo-induced ferroptosis in non-irradiated B cells. Moreover, CDKN1A inhibition partially counteracted the suppressive effect of miR-34a-5p knockdown on non-irradiated B cell ferroptosis.

**Conclusions:**

Our findings suggest that irradiated exosomal miR-34a-5p promotes non-irradiated B cell ferroptosis through CDKN1A, uncovering a novel mechanism for RIBE and offering a new therapeutic target for radioprotection.

## Introduction

Ionizing radiation has been applied in a wide range of fields, including treatment procedures and industrial applications. Despite the benefits, long-term or high-dose exposure to ionizing radiation can have harmful effects on the surgeon and staff [1]. Prolonged or high doses of radiation exposure may lead to a decrease in the number of lymphocytes, a phenomenon known as lymphocytopenia leading to the risk of infection [2]. Different lymphocyte subsets have different sensitivity to radiation, among which B cells present well-known high radiation sensitivity [3]. The radiation-induced bystander effect (RIBE) refers to numerous biological effects in non-irradiated cells, which are triggered by receiving bystander signals released from irradiated cells [4]. These effects include gene instability, gene mutations, chromosomal aberrations, and apoptosis. Therefore, studying the RIBE in B cells, key players in the immune system, helps deepen our understanding of how radiation signals transmitted via intercellular communication to non-irradiated cells, thereby inducing biological effects, providing vital clues for improving radiotherapy safety.

In addition to damaging cellular DNA and causing double-strand breaks, radiation also ionizes the cytoplasm and mitochondria, leading to the generation of a large amount of reactive oxygen species, iron imbalance and lipid peroxidation which are related to ferroptotic damage [5]. As a highly iron-dependent form of cell death, ferroptosis clearly distinguishes itself from other types of regulated cell death [6]. Research has found that radiation is closely related to ferroptosis [7]. Radiation can boost the activity of ACSL4, causing higher levels of lipid peroxidation and consequently ferroptosis [8]. Sphingosine 1-phosphate mitigates radiation-triggered ferroptosis in ovarian granulosa cells through the upregulation of GPX4 [9]. Radiation-triggered bone marrow hemorrhage led to ferroptosis in granulocyte-macrophage hematopoietic progenitor cells [10]. Ferroptosis has potential applications in the treatment of B lymphocyte-associated immune diseases, like immune deficiency and diffuse large B-cell lymphoma [11]. Nevertheless, the evidence supporting a connection between radiation and B cell ferroptosis is currently insufficient.

Exosomes, nanoscale extracellular vesicles secreted by nearly all mammalian cells, serve a vital function in intercellular communication through the transfer of bioactive molecules between the originating cell and the receiving cell [12]. A growing body of evidence confirms that exosome-derived miRNAs exert a crucial role in RIBE [13]. Exosomal delivery of miR-769-5p aggravated the bystander effect caused by ultraviolet exposure via inhibiting TGFBR1 in human skin fibroblasts [14]. Exosomes assisted in the transportation of miR-4655-3p between cells, which enhanced the ultraviolet induced RIBE in the bystander cells via restraining the expression of E2F2 [15]. Radiation-induced human bronchial epithelial cell exosomal miR-7-5p triggered bystander autophagy [16]. However, whether irradiated exosome-mediated miRNA transfer plays a role in RIBE of B cells remains still indistinct.

In this study, we aim to ascertain whether B cell-derived exosomal miRNA regulates RIBE through ferroptosis. We isolated and characterized exosomes from irradiated and non-irradiated B cells. Key miRNAs involved in regulating ferroptosis were identified through small RNA sequencing. Cell experiments were conducted to explore the impact of irradiated B cell-derived exosomal miRNA on the ferroptosis of non-irradiated B cells. Our research provides a new perspective for understanding the RIBE in B cells.

## Materials and methods

### Cell culture and radiation treatment

Human peripheral blood B lymphocyte IM-9 cell line (iCell-h517) and AHH-1 cell line (YS2039C) were purchased from iCellbioscience and Shanghai Yaji Biotechnology Co., Ltd., respectively. IM-9 cells were maintained in RPMI 1640 medium (Corning, 10-040-CVRC), to which 10 % fetal bovine serum (FBS) and 1 % penicillin streptomycin (P/S) were added. AHH-1 cells were cultured in DMEM containing 10 % FBS and 1 % P/S. These cells were incubated at 37 °C in a humidified environment with 5 % CO2.

For radiation treatment, IM-9 cells were grown to the logarithmic phase and irradiated with 0 Gy, 0.1 Gy, 0.2 Gy, and 2 Gy using a irradiator (Radsource, RS2000Pro-225).

### Exosome isolation

IM-9 cells were grown to the logarithmic phase. Following three washes with PBS, the cells were further incubated in serum-free media for 48 h. To remove cell pellets, the collected culture medium was centrifuged at 300*g* for 10 min at 4 °C. Supernatants were centrifuged at 2,000*g* for 10 min at 4 °C followed by 10,000 g for 30 min at 4 °C. Next, supernatants were centrifuged at 120,000 g for 2 h at 4 °C to pellet exosomes. To obtain the exosome suspension for subsequent experiments, we added pre-cooled PBS to the isolated exosome precipitate, then mix it thoroughly until the exosomes are completely dissolved.

### Nanoparticle tracking analysis (NTA)

NTA was conducted using ZetaView PMX 110 (Particle Metrix, Meerbusch, Germany). Cleaning of the sample pool was performed using deionized water, followed by calibration of the instrument with polystyrene microspheres. After washing the sample pool with PBS buffer, the sample for detection was added. The ZetaView 8.04.02 SP2 was used for particle size distribution and particle concentration. Particle concentration (particles/mL) was automatically calculated by the software based on the number of particles detected per frame and the effective measurement volume.

### Transmission electron microscopy (TEM)

The vesicles’ morphology was characterized by a TEM. The sample was dropped onto a copper mesh and the excess liquid was sucked away. The copper mesh was then added with phosphotungstic acid. Observations of all samples were conducted via the TEM (JEM-1200EX, Japan) at a voltage of 100 kV.

### Western blot

Cells and exosomes were lysed in RIPA buffer and the proteins underwent separation via SDS-PAGE. The proteins were then transferred to polyvinylidene difluoride (PVDF) membranes. Following a 1-h blocking step with 5 % non-fat milk, the membranes were incubated with primary antibodies against TSG101 (1:5,000, ab125011, Abcam), ALIX (1:10,000, 12422-1-AP, Proteintech), SLC7A11 (1:2,000, 26864-1-AP, Proteintech), GPX4 (1:1,000, sc-166570, Santa cruz), FTH1 (1:5,000, 11682-1-AP, Proteintech), CDKN1A (1:20,00, 82669-2-RR, Proteintech), and GAPDH (1:15,000, 60004-1-Ig, Proteintech) overnight at 4 °C. On the following day, incubation with secondary antibodies HRP-conjugated Goat Anti-Rabbit IgG (1:5,000, SA00001-2, Proteintech) or HRP-conjugated Goat Anti-mouse IgG (1:5,000, SA00001-2, Proteintech) was performed for 1 h at room temperature, after which visualization of the membranes was performed using an ECL chemiluminescence system.

### Small RNA sequencing (RNA-seq)

Total RNA was segregated from non-irradiated B cell derived exosomes (non-IR-exo) and irradiated B cell derived exosomes (IR-exo) with TRIzol reagent (Invitrogen, USA). RNA quality and concentration were assessed by utilizing a NanoDrop ONE spectrophotometer (Thermo Fisher Scientific, USA), while integrity was confirmed via 2 % agarose gel electrophoresis. Small RNA libraries were prepared using the Multiplex Small RNA Library Prep Kit (USA). Briefly, RNA fragments were ligated with adapters at both ends, followed by cDNA synthesis and PCR amplification. The resulting PCR products were analyzed by 8 % SDS-PAGE. Sequencing was implemented on the Illumina HiSeq X 10 platform (USA). For miRNA analysis, we compared the filtered clean reads to the human miRNA database to obtain the expression status of miRNA. To account for differences in sequencing depth and library size, raw counts were normalized using the median-of-ratios method implemented in the DESeq2 R package. Differentially expressed miRNAs were identified based on the criteria of |log2 (fold change)|>1 and a p-value<0.05. Gene ontology (GO) and Kyoto Encyclopedia of Genes and Genomes (KEGG) pathway enrichment analyses for the target mRNAs of identified miRNA were conducted using a p-value threshold of 0.05.

### Screening of candidate miRNAs regulating ferroptosis

GSE18760 and GSE97000 were acquired from the GEO database. In GSE18760, we compared the differentially expressed genes between bystander IMR90 cells and non-radiated IMR90 cells 4 h after irradiation. In GSE97000, we compared the differentially expressed genes between normal human lymphocytes and human lymphocytes 24 h after 2 Gy *γ*-ray irradiation. We took the intersection of the target genes that upregulate miRNA and the gene sets that inhibit ferroptosis. The ferroptosis suppressor genes in the intersection are then intersected with the genes down-regulated after radiation from GSE18760 and GSE97000. The upregulated miRNAs corresponding to the final intersection genes are candidate miRNAs.

### Quantitative reverse transcription-polymerase chain reaction (qRT-PCR)

Total RNA was isolated from IM-9 cells with TRIzol reagent. Reverse transcription from extracted RNA was performed using PrimeScript RT reagent Kit (K1622, Thermo). qRT-PCR was executed with SYBR GreenER qPCR SuperMix Universal (Roche) as per the manufacturer’s guidelines. Cycling conditions involved an initial step at 95 °C for 10 min, followed by 45 cycles of 95 °C for 15 s and 60 °C for 60 s. Relative expression of miR-34a-5p, miR-138-5p, miR-194-5p, miR-625-3p, SLC7A11, GPX4, FTH1, and CDKN1A was determined by the 2ˆ(-ΔΔCt) method, with normalization to U6 or glyceraldehyde 3-phosphate dehydrogenase (GAPDH). The sequences of primers were listed in Table S1.

### Cell transfection

miR-34a-5p mimics, mimics NC, miR-34a-5p inhibitor and inhibitor NC, si-NC, and si-CDKN1A were obtained from GenePharma (Shanghai, China). The transfection of B cells with miR-34a-5p mimics, mimics NC, miR-34a-5p inhibitor, inhibitor NC, si-NC, and si-CDKN1A was performed using the Lipofectamine 2000 reagent (Invitrogen), in accordance with the guidelines provided by the manufacturer.

### Cell counting kit-8 (CCK-8) assay

To measure cell viability, a CCK-8 kit (Beyotime, China) was utilized. Cells were seeded into 96-well plates at a density of 2000 cells per well. B cells were incubated with PBS, non-irradiated exosomes, irradiated exosomes and miR-34a-5p knockdown irradiated exosomes. After adding CCK-8 solution to each well and incubating for 2 h, absorbance was measured at 450 nm. Each condition was assayed in six replicates.

### Detection of lactate dehydrogenase (LDH) activity

The activity of LDH was measured by the LDH assay kit (Solarbio, BC0680) using the protocol supplied by the manufacturer. In short, cells were inoculated in 96-well plates with a density of 2 × 10^3^ cells/well. B cells were then incubated with PBS, unradiated exosomes, radiated exosomes and miR-34a-5p knockdown radiated exosomes. After incubation for 48 h, the samples underwent centrifugation (500*g*, 5 min) to collect the supernatant. The absorbance was quantified at 450 nm via a microplate reader (Thermo Fisher Scientific, USA).

### Measurement of lipid peroxidation

B cells were seeded into 12-well dishes and treated with PBS, unradiated exosomes, radiated exosomes and miR-34a-5p knockdown radiated exosomes for 48 h. Next, B cells were incubated with BODIPY-581/591C11 storage solution for 60 min at 37 °C. After being washed once with PBS, the cells were incubated with 1 × Hoechst 33,342 live cell staining solution (C1029, Beyotime) for 15 min at ambient temperature. Microscopy was performed using a fluorescence microscope (CKX53, OLYMPUS).

### Dual-luciferase reporter assay

The 3′-UTR fragment of CDKN1A containing the predicted miR-34a-5p binding site was inserted into the pmirGLO or psiCHECK-2 vector to generate wild-type (WT) and mutant (MUT) luciferase reporters. HEK293T cells were co-transfected with reporter plasmids and miR-34a-5p mimics or negative control using Lipofectamine 3000. After 48 h, luciferase activity was measured with the Dual-Luciferase^®^ Reporter Assay System (Promega).

### Statistical analysis

Each experiment was conducted in triplicate. Data are expressed as the mean ± standard deviation (SD). Statistical analysis was performed using GraphPad Prism 9.0. Differences between two groups were assessed by Student’s *t*-test, while comparisons among multiple groups utilized one-way ANOVA followed by Tukey’s post hoc test. Statistically significant differences were indicated as follows: * p<0.05, ** p<0.01.

## Results

### Characterization of exosomes from irradiated B cells

**Figure 1:**
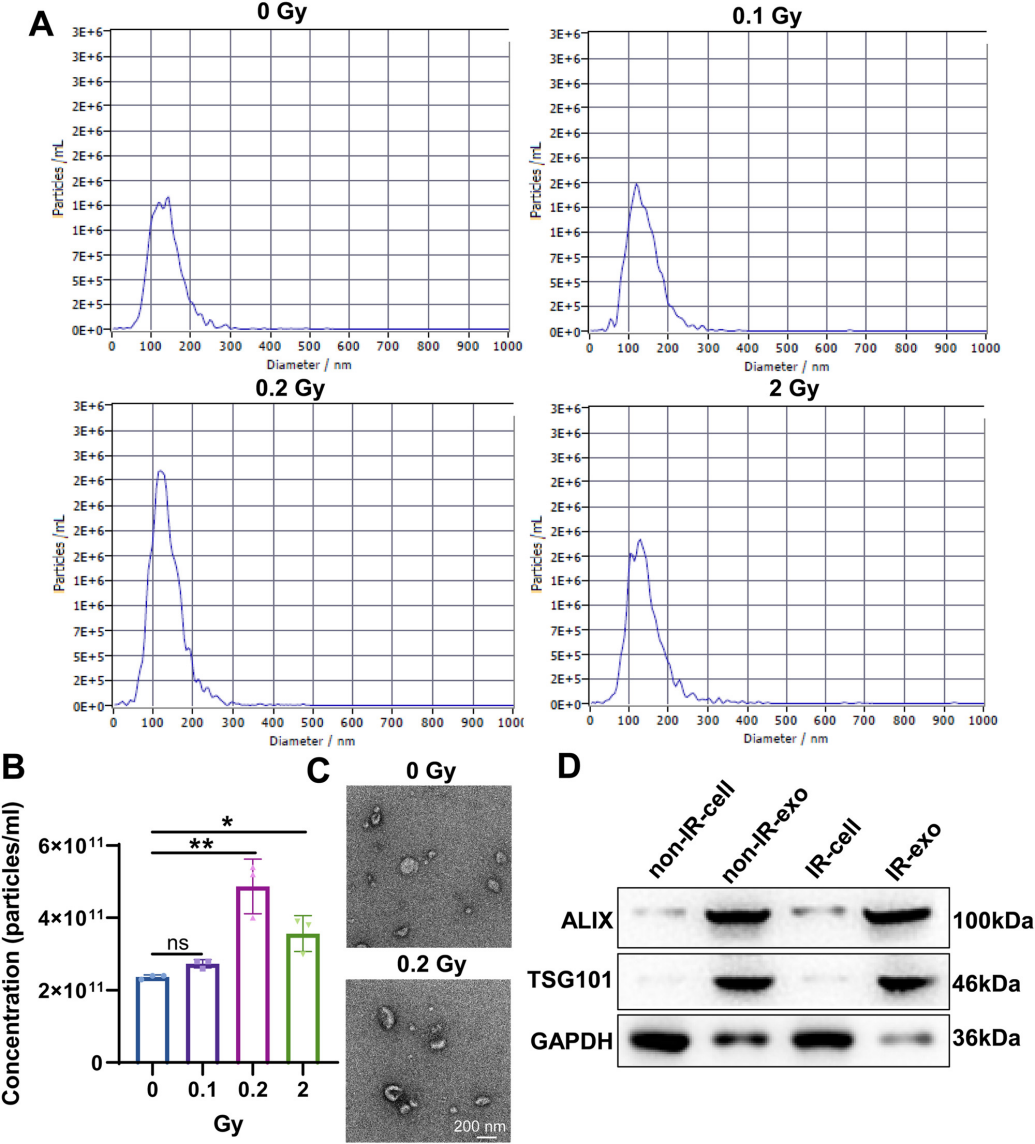
Identification of exosomes from irradiated B cells. (A) Nanoparticle tracking analysis was utilized to evaluate the number and size distribution of particles. (B) Concentration of exosomes from B cells treated with radiation of different doses. (C) Representative image of the exosomes by using transmission electron microscopy. (D) western blot analysis of exosome markers ALIX and TSG101 expression in isolated exosomes and cell lysates. The data are depicted as the mean ± standard deviation (SD). n=3. ns, no significance. *, p<0.05; **, p<0.01.

To determine the radiation dose of B cells, IM-9 cells were irradiated with various does of radiation (0, 0.1, 0.2, 2 Gy). The concentration and size of exosomes were assessed by NTA. The NTA results uncovered that the diameters of the isolated particles were 50–150 nm (Figure 1A). NTA results showed that exosome concentrations were increased slightly at 0.1 Gy, but were significantly enhanced at 0.2 and 2 Gy (Figure 1B). Since exosome concentration was highest and most significantly different at 0.2 Gy, we chose 0.2 Gy as the optimal dose for our follow-up experiments. TEM showed that non-IR-exo and IR-exo exhibited typical cup-shaped morphology, suggesting the successful isolation of exosomes (Figure 1C). Furthermore, western blot uncovered an enriched expression of exosomal markers, including TSG101 and ALIX in non-IR-exo and IR-exo (Figure 1D). Overall, we successfully isolated non-IR-exo and IR-exo.

### Irradiated exosomes promotes ferroptosis of non-irradiated B cells

**Figure 2:**
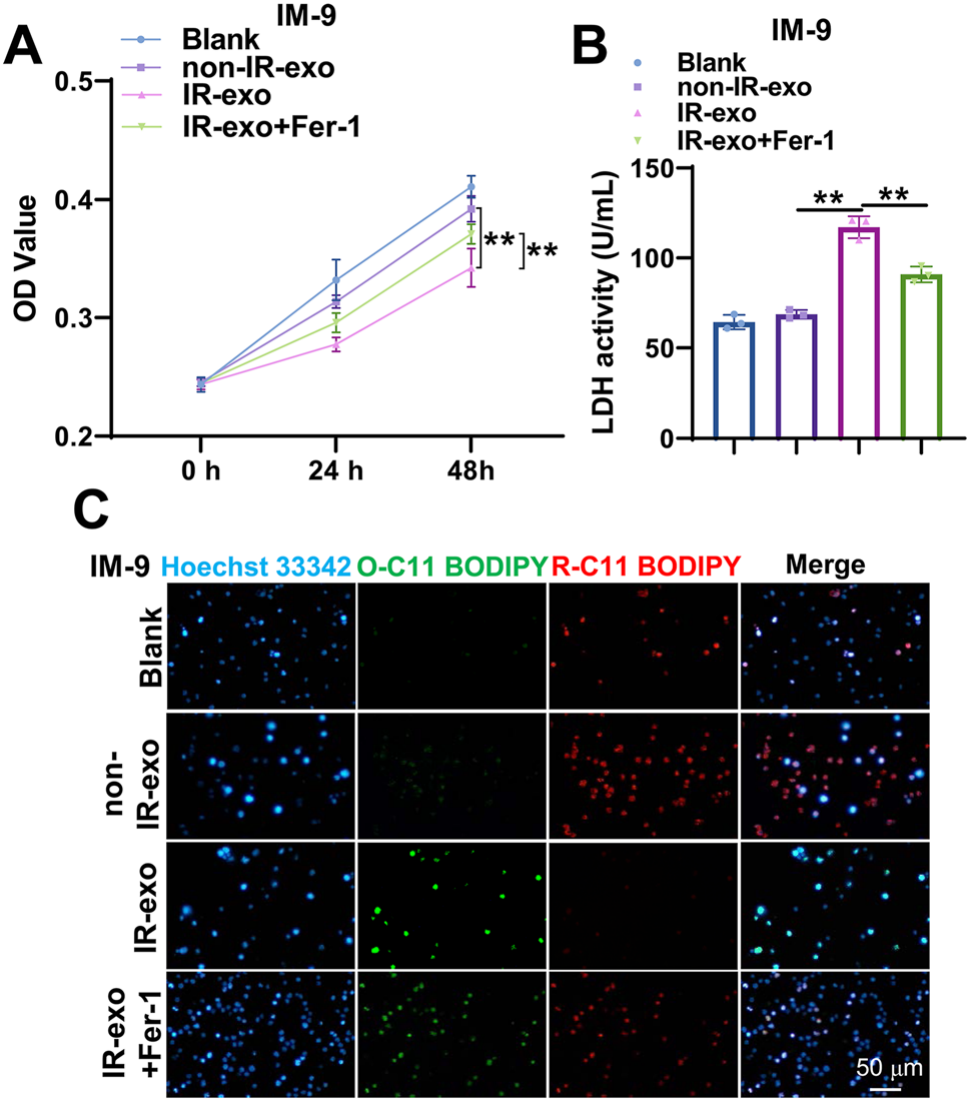
IR-exo induces ferroptosis in B cells. (A) The CCK-8 assay was applied to detect the effect of IR-exo and Fer-1 on cell proliferation. (B) The LDH detection assay was applied to evaluate the effect of IR-exo and Fer-1 on LDH activity. (C) The BODIPY 581/591 C11 staining was utilized to determine the effect of IR-exo and Fer-1 on lipid peroxidation. All experiments were conducted with n=3, except for the CCK-8 assay, which was conducted with n=6. The data are depicted as the mean ± standard deviation (SD). **, p<0.01.

To probe into the role of IR-exo in ferroptosis of B cells, we treated IM-9 cells with IR-exo and Fer-1. CCK-8 assays showed that IR-exo significantly suppressed the proliferation of IM-9 cells, an effect that was reversed by Fer-1 (Figure 2A). An LDH detection assay revealed markedly elevated LDH activity in the IR-exo group. This elevation was significantly attenuated after Fer-1 treatment (Figure 2B). Furthermore, assessment of lipid peroxidation using the BODIPY 581/591 C11 probe showed that IR-exo induced an increase in lipid peroxidation, which was neutralized by Fer-1 treatment (Figure 2C). Overall, IR-exo trigger non-irradiated B cell ferroptosis.

### Expression profiling of miRNAs in irradiated B cell-derived exosomes

**Figure 3:**
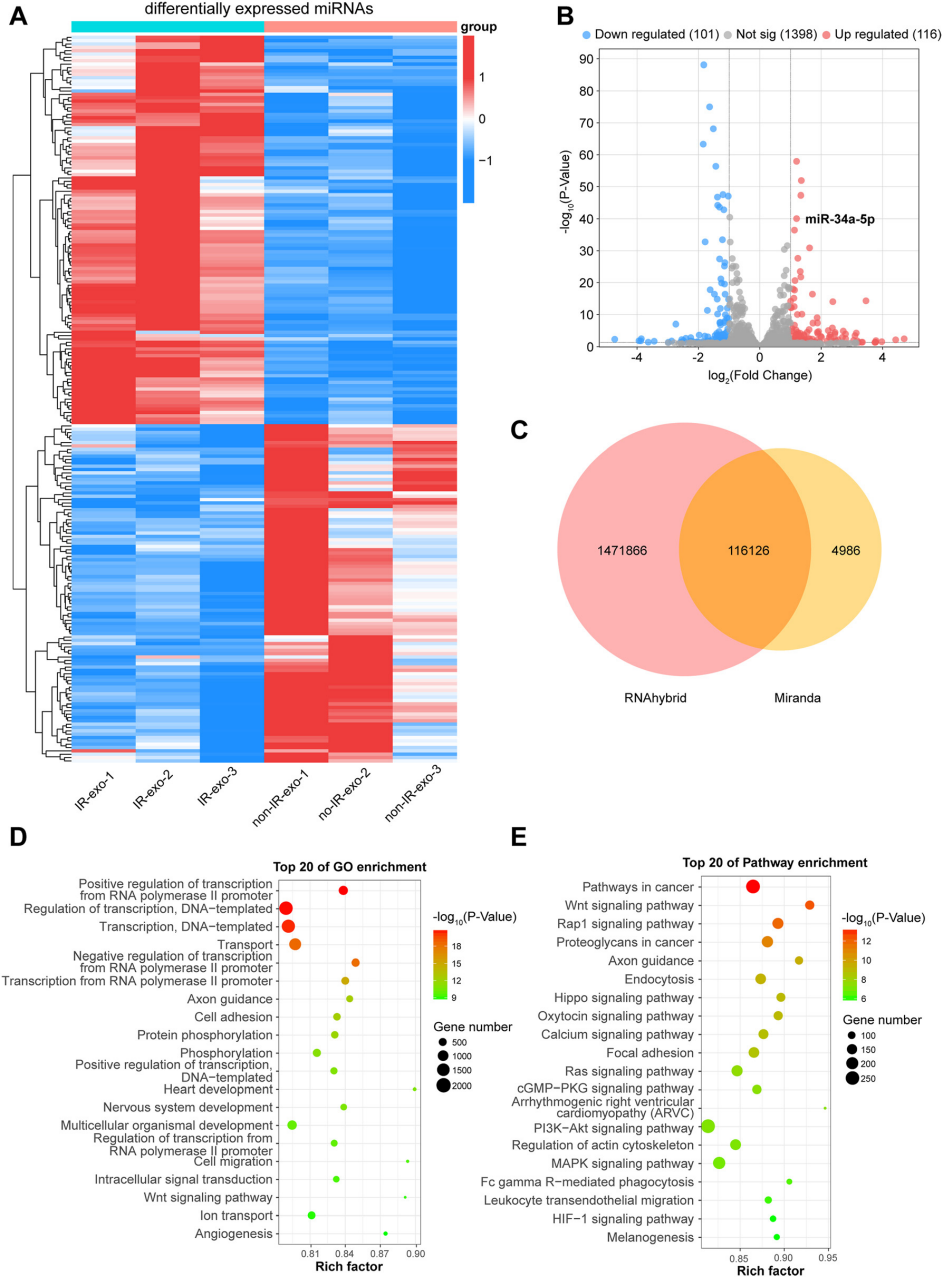
The expression profile of miRNAs in the non-IR-exo and IR-exo groups. (A, B) Differential patterns of miRNA expression between the non-IR-exo and IR-exo groups as shown by heatmap (A) and volcano map (B) (n=3). (C) The potential target genes for differentially expressed miRNAs from RNAhybrid and miRanda were intersected using a venn diagram. (D, E) GO (D) and KEGG (E) analysis of target genes of differentially expressed miRNAs between the non-IR-exo and IR-exo groups (n=3).

To determine the function of radiation on the expression of small RNAs in B cell derived exosomes, small RNA sequencing in exosomes derived from IM-9 cells before and after irradiation was performed. The expression patterns of miRNAs were compared between non-IR-exo and IR-exo groups. Compared to the non-IR-exo group, 217 differentially expressed miRNAs were acquired in the IR-exo group, including 116 up-regulated and 101 down-regulated miRNAs (Figure 3A and B). The top 10 most upregulated and downregulated miRNAs between the non-IR-exo and IR-exo groups were shown in Table S2. Potential target genes of the miRNAs were predicted via RNAhybrid and miRanda software. A total of 116,126 target genes that were predicted by both algorithm (Figure 3C). GO analysis manifested that target genes of differentially expressed miRNAs were chiefly involved in cell migration, intracellular signal transduction, and ion transport (Figure 3D). KEGG analysis indicated that these target genes primarily participated in Wnt, Hippo, and MAPK signaling pathways (Figure 3E). Overall, these results indicate that radiation leads to differential expression of miRNAs in B cell-derived exosomes.

### miR-34a-5p is the target of irradiated B cell-derived exosomes

**Figure 4:**
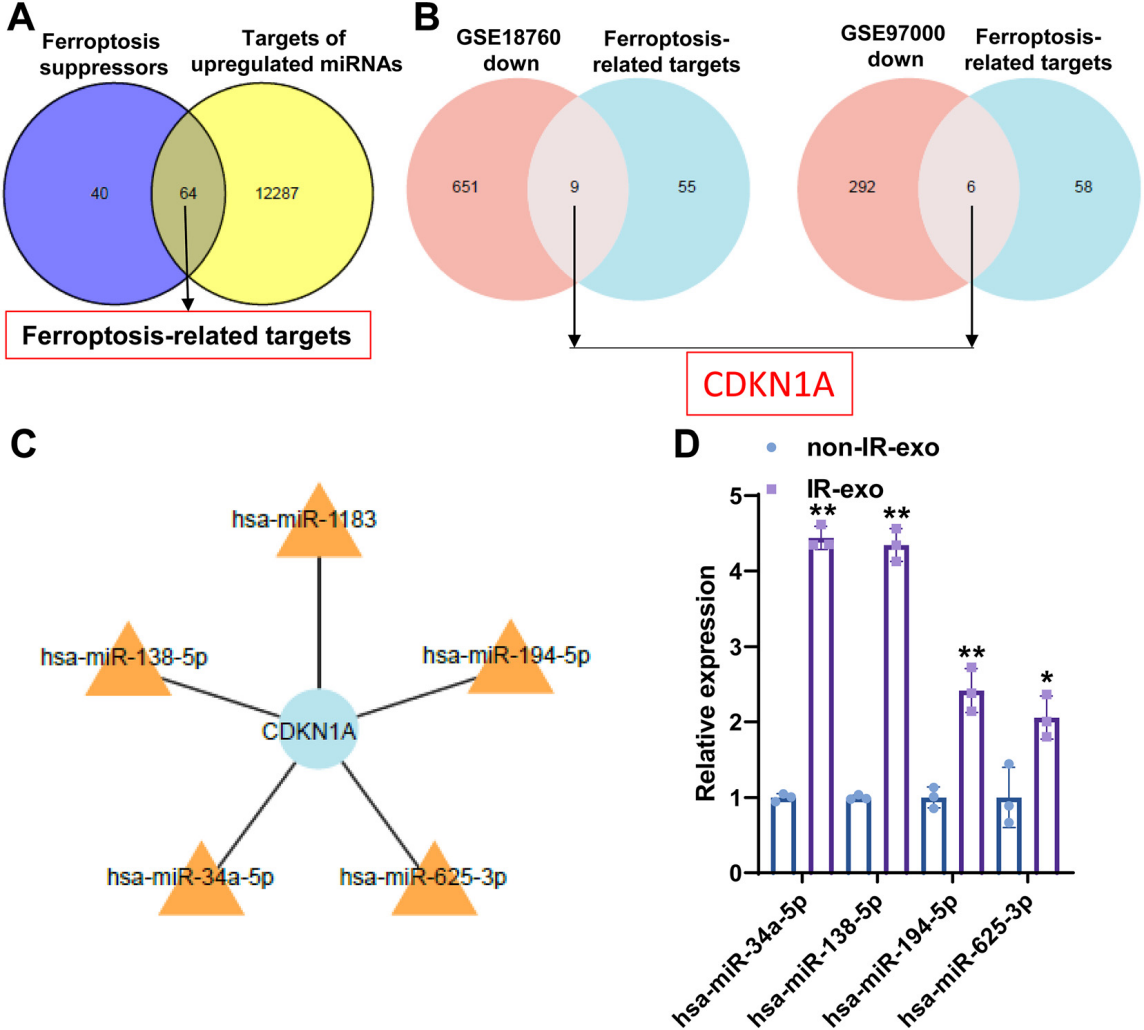
miR-34a-5p is upregulated in IR-exo. (A) An overlap between target genes of upregulated miRNAs from small RNA sequencing and ferroptosis suppressors from the FerrDB database. (B) Venn diagram showing the shared downregulated genes between 64 ferroptosis inhibition-related genes and downregulated genes from GSE18760 and GSE97000. (C) The miRNA-target genes network. (D) qRT-PCR was employed to detect the levels of candidate miRNAs in the non-IR-exo and IR-exo groups (n=3). The data are depicted as the mean ± standard deviation (SD). *, p<0.05; **, p<0.01.

Our study revealed that target genes of differentially expressed miRNAs were enriched in Wnt, Hippo , and MAPK signaling pathways. These pathways were discovered to be closely related to ferroptosis [17], [18], [19], suggesting that IR-exo miRNAs may regulate ferroptosis by targeting ferroptosis-related genes. Subsequently, to determine key miRNAs involved in ferroptosis, we firstly screened genes related to ferroptosis from the FerrDB database. By intersecting the target genes of upregulated miRNAs with ferroptosis suppressors, we obtained 64 common target genes (Figure 4A). We then took the intersection of these 64 genes with downregulated genes from GEO datasets (GSE18760 and GSE97000). This intersection identified nine overlapping genes in GSE18760 and six in GSE97000 (Figure 4B). Notably, CDKN1A was the only gene common to both sets. Afterwards, five differentially expressed miRNAs with high fold change, which targeted CDKN1A, were selected for the next study, including miR-138-5p, miR-34a-5p, miR-625-3p, miR-194-5p, and miR-1183 (Figure 4C). Subsequent qRT-PCR validation excluded miR-1183 due to an abnormal melting curve. Quantification of the remaining four miRNAs revealed that miR-34a-5p was not only significantly upregulated in the IR-exo group but also exhibited the greatest fold change (Figure 4D). Consequently, miR-34a-5p was selected for further functional validation.

### Irradiated exosomal miR-34a-5p promotes ferroptosis of non-irradiated B cells

**Figure 5:**
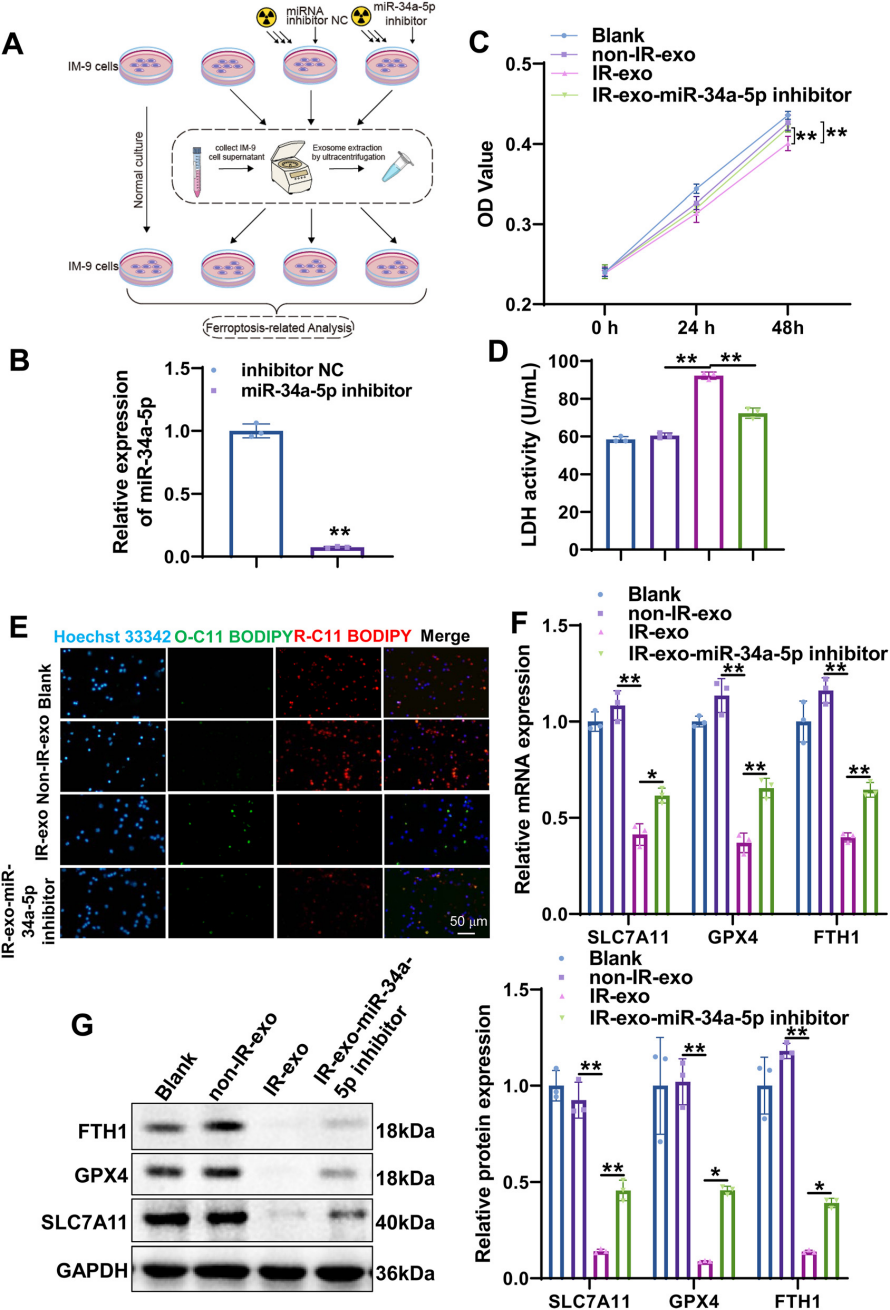
Irradiated exosomal miR-34a-5p promotes ferroptosis of B cells. (A) Flowchart of the effect of irradiated exosomal miR-34a-5p knockdown on ferroptosis in IM-9 cells. (B) Knockdown efficiency of miR-34a-5p was verified by qRT-PCR. (C) The impact of irradiated exosomal miR-34a-5p knockdown on cell viability of IM-9 cells was assessed by the CCK-8 assay. (D) Lactate dehydrogenase (LDH) activity was examined in IM-9 cells incubated with miR-34a-5p knockdown IR-exo by the LDH detection kit. (E) Representative images for the BODIPY C-11 assay were acquired by confocal laser microscopy. (F, G) qRT-PCR (F) and western blot (G) were utilized to assess the expression of ferroptosis related genes in IM-9 cells incubated with miR-34a-5p knockdown IR-exo. All experiments were conducted with n=3, except for the CCK-8 assay, which was conducted with n=6. The data are depicted as the mean ± standard deviation (SD). *, p<0.05; **, p<0.01.

To further ascertain the role of irradiated exosomal miR-34a-5p in ferroptosis of B cells, we detected the effect of miR-34a-5p knockdown exosomes on ferroptosis indicators *in vitro* (Figure 5A). We transfected the irradiated IM-9 cells with miR-34a-5p inhibitor or inhibitor NC. qRT-PCR showed that miR-34a inhibitor drastically decreased the level of miR-34a-5p in irradiated exosomes (Figure 5B). Subsequently, the CCK-8 assay was used to monitor IM-9 cell proliferation. As shown in Figure 5C, IR-exo significantly inhibited IM-9 cell proliferation, whereas miR-34a-5p knockdown reversed the proliferation inhibition induced by IR-exo. LDH detection assay indicated that the LDH activity in the IR-exo group was markedly increased, while the miR-34a knockdown IR-exo group exhibited significantly lower LDH activity than the IR-exo group (Figure 5D). We then measured lipid peroxidation with BODIPY 581/591 C11 fluorescence probe. Lipid peroxidation was significantly enhanced in the IR-exo group, which was counteracted by miR-34a-5p knockdown (Figure 5E). In addition, to further detect the effect of IR-exo with miR-34a-5p knockdown on ferroptosis in IM-9 cells, the expression of ferroptosis-related markers SLC7A11, GPX4, and FTH1 was assessed by qRT-PCR and western blot. The results uncovered that IR-exo significantly inhibited the expression of SLC7A11, GPX4, and FTH1 mRNA and protein, while interference of miR-34a-5p promoted the expression of these molecules in IR-exo (Figure 5F and G). Furthermore, IR-exo promoted ferroptosis in non-irradiated AHH-1 cells, as evidenced by decreased cell viability, increased LDH activity and elevated lipid peroxidation (Figure S1A–C). However, inhibition of miR-34a-5p in IR-exo reversed these effects. These findings suggest that irradiated exosomal miR-34a-5p accelerated ferroptosis of non-irradiated B cells.

### CDKN1A participates in irradiated exosomal miR-34a-5p-mediated non-irradiated B cell ferroptosis

**Figure 6:**
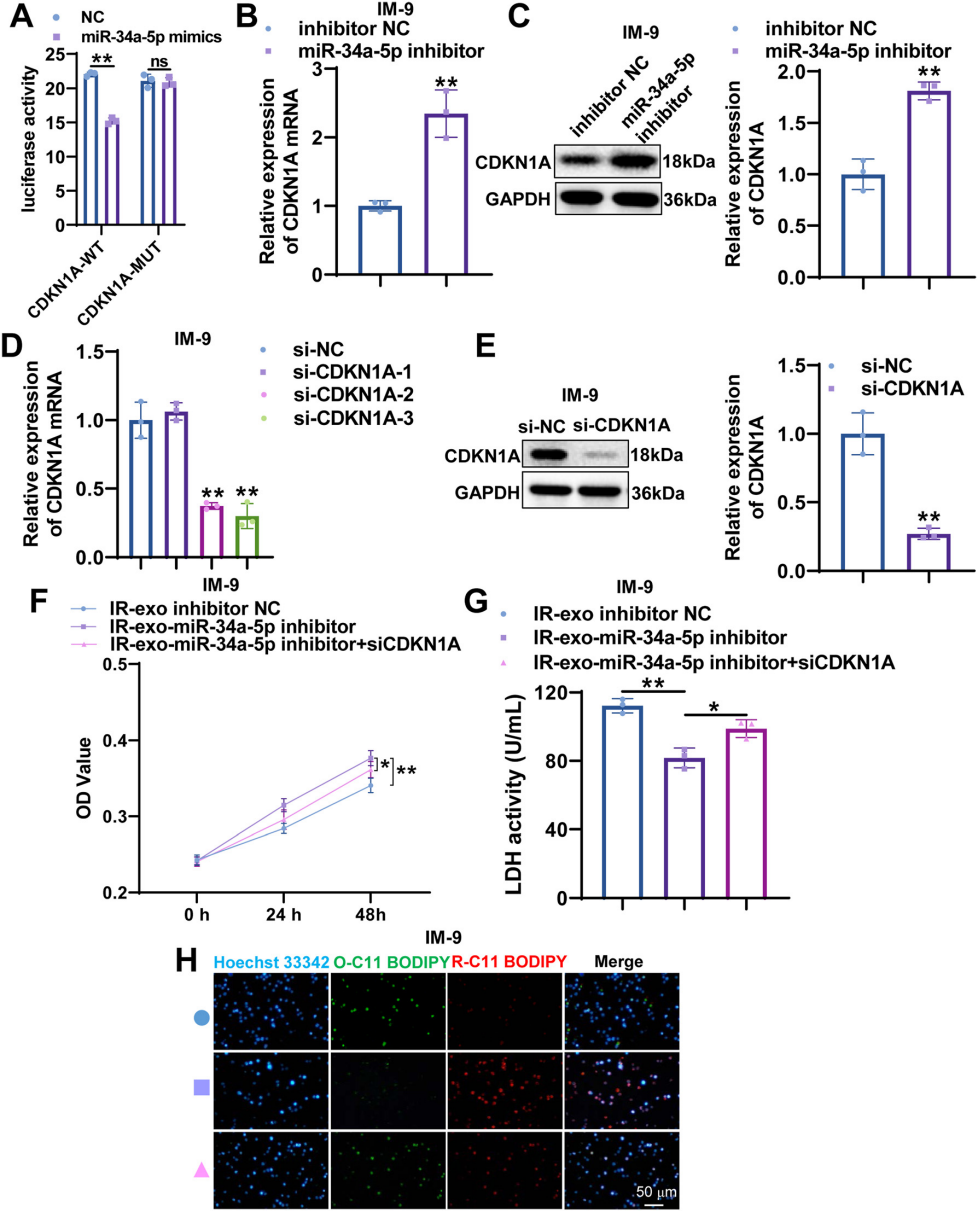
miR-34a-5p directly targets CDKN1A to promotes ferroptosis of B cells. (A) The interaction between miR-34a-5p and CDKN1A was verified using a dual-luciferase reporter assay. (B, C) CDKN1A mRNA and protein levels were detected by qRT-PCR and western blot in IM-9 cells after miR-34a-5p inhibition. (D, E) qRT-PCR and western blot analysis CDKN1A knockdown efficiency in IM-9 cells. (F–H) The abilities of cell viability, LDH activity, and lipid peroxidation in IM-9 cells treated with miR-34a-5p inhibitor NC, miR-34a-5p inhibitor, or the combination of miR-34a-5p inhibitor and siCDKN1A. All experiments were conducted with n=3, except for the CCK-8 assay, which was conducted with n=6. The data are depicted as the mean ± standard deviation (SD). *, p<0.05; **, p<0.01.

To verify CDKN1A is regulated by miR-34a-5p, the luciferase reporter assay was performed. The results showed that miR-34a-5p mimics markedly reduced the relative luciferase activity in cells transfected with the wild-type CDKN1A 3′UTR, whereas no impact on luciferase activity was found in cells transfected with the mutant CDKN1A 3′UTR (Figure 6A). The results of qRT-PCR and western blot indicated that the mRNA and protein expression of CDKN1A in IM-9 cells was significantly upregulated after miR-34a-5p knockdown (Figure 6B and C). To further investigate the functional correlation between miR-34a-5p and CDKN1A in non-irradiated B cell ferroptosis, the rescue experiment was conducted. The knockdown efficiency of CDKN1A was confirmed by qRT-PCR and western blot (Figure 6D and E). CDKN1A inhibition counteracted the anti-ferroptotic effects induced by miR-34a-5p downregulation in IR-exo, as indicated by diminished cell viability, augmented LDH activity, and accumulated lipid peroxidation in non-irradiated IM-9 cells (Figure 6F–H). Together, these findings indicate that irradiated exosomal miR-34a-5p promotes ferroptosis in non-irradiated B cells by inhibiting CDKN1A.

## Discussion

Although precision radiotherapy technology is continuously advancing, radiation-induced damage remains unavoidable. B cells are a key part of the immune system and are mainly responsible for producing antibodies to deal with pathogens. However, B cells are highly sensitive to radiation and are prone to radiation damage, which can affect immune function [20]. Consequently, exploring the mechanisms underlying the radiation sensitivity of B cells holds great importance for guiding radiotherapy. In the present study, we confirmed that miR-34a-5p in IR-exo was a key molecule regulating ferroptosis through small RNA sequencing. miR-34a-5p was significantly enhanced in IR-exo. Cell experiments revealed that knockdown of miR-34a-5p could reverse ferroptosis in non-irradiated B cells induced by IR-exo. Mechanistically, irradiated exosomal miR-34a-5p induced non-irradiated B cell ferroptosis via downregulating CDKN1A (Figure S2). Overall, the role and mechanism of exosomal miR-34a-5p in the RIBE provides a new perspective for understanding the impact of radiation on non-irradiated cells, and also offers potential new strategies for radiotherapy protection.

Exosomes play a vital part in the RIBE. Exosomes regulate the biological behavior of unradiated cells by carrying signal molecules such as nucleic acids, proteins and lipids to transmit information between cells [21]. A study found that radiation-induced exosomes mediate RIBE by delivering mitochondrial DNA [22]. Another study pointed out that the exosomes released by cells after radiation changed the composition of their contents, thereby affecting the proliferation, migration and apoptosis of recipient cells. These alterations further led to resistance to radiation therapy [23]. Extracellular vesicle release triggered by ionizing radiation enhanced viability, migration of neuroblastoma cells [24]. Exosome-mediated RIBE induced replication stress in recipient cells [25]. These studies have primarily focused on phenotypes such as stress, apoptosis, and migration. However, whether exosome-mediated RIBE affects ferroptosis in recipient cells remains unclear. In our study, we discovered that IR-exo promoted ferroptosis of B cells, as evident by increased LDH activity and lipid peroxidation, which was reversed by Fer-1. Therefore, Targeting ferroptosis opens up a novel avenue for the study of RIBE.

In the occurrence mechanism of the RIBE, exosomal miRNA is regarded as a key signal transduction medium [13]. Radiation can alter the expression profile of miRNAs within cells, and these miRNAs are subsequently released outside the cells through exosomes, thereby mediating the biological responses of unradiated cells [26]. For example, radiation-induced exosomal miR-7-5p promoted autophagy in non-irradiated human bronchial epithelial cells via the EGFR signaling pathway [16]. The expression of miR-4655-3p in exosomes was elevated under ultraviolet irradiation, and the upregulation of miR-4655-3p enhanced the ultraviolet-induced bystander effect in human skin fibroblasts [15]. The miR-1246 expression in exosomes secreted by irradiated BEP2D cells was increased and miR-1246 knockdowne inhibited the proliferation of non-irradiated cells [27]. In our study, the expression of miR-34a-5p in IR-exo was significantly upregulated. miR-34a-5p has been reported to play a core role in ferroptosis of various cells. For example, in epilepsy, miR-34a-5p contributed to hippocampal neuronal ferroptosis [28]. Inhibition of miR-34a-5p attenuated CdCl_2_-induced PC12 cell ferroptosis [29]. In agreement with these findings, we discovered that silence of miR-34a-5p offset the promotive effect of IR-exo on ferroptosis of B cells. Together, the discovery of exosomal miR-34a-5p provides a new perspective for understanding the mechanism of RIBE in B cells.

In the clinical context, miR-34a-5p is broadly acknowledged as a tumor-suppressive miRNA, with its expression levels being strongly linked to patient prognosis in multiple cancer types. For instance, In colorectal cancer, miR-34a-5p expression had a positive relation with disease-free survival [30]. In head and neck squamous cell cancer, low miR-34a-5p expression correlates with advanced TNM stage [31]. Our current study found that miR-34a-5p in IR-exo induces ferroptosis in non-irradiated B cells by targeting CDKN1A. These findings suggest that miR-34a-5p may play a role in modulating the radiotherapy-associated immune microenvironment. However, its precise clinical relevance in governing radiotherapy response and immune regulation remains to be elucidated and warrants further investigation.

Although exosomes have been reported to participate in RIBE [32], research on the specific regulatory mechanism of the miRNAs they carry in immune cells remains scarce. In our study, CDKN1A was a predicted target gene of miR-34a-5p through small RNA sequencing analysis. CDKN1A is an important cell cycle regulatory protein and related to ferroptosis inhibition [33]. Research indicates that miR-6077 induced cell cycle arrest and ferroptosis by targeting CDKN1A/KEAP1 in lung adenocarcinoma [34]. CDKN1A knockdown alleviated cisplatin-induced cell injury by mitigating oxidative stress and ferroptosis in HK-2 and NRK-52E cells [35]. CDKN1A was also discovered to exert a suppressive effect on ferroptosis in renal tubular epithelial cells [36]. In our study, CDKN1A siliencing could reverse the inhibitory effect of knockdown of exosomal miR-34a-5p on ferroptosis of B cells, indicating that CDKN1A serves as a critical downstream effector through which miR-34a-5p regulates B cell ferroptosis.

Our research indicates that irradiated exosomal miR-34a-5p induced ferroptosis in non-irradiated B cells via CDKN1A, providing a new direction for understanding the molecular mechanism of RIBE. However, to translate this *in vitro* finding into *in vivo* or clinical settings, several key issues still need to be considered. Firstly, it is necessary to verify in animal models whether exosomal miR-34a-5p circulates *in vivo* and induces bystander responses. Secondly, it is necessary to evaluate whether plasma exosomal miR-34a-5p can be used as a biomarker for measuring individual radiation sensitivity. The solutions to these problems will provide brand-new strategies for the development of new radiation protectants to reduce the damage of radiotherapy to normal tissues, thereby improving the quality of life and treatment safety of patients.

In conclusion, our study found that miR-34a-5p in IR-exo was significantly upregulated and could facilitate ferroptosis in non-irradiated B cells via inhibiting CDKN1A. Our findings revealed a new mechanism by which IR-exo regulate B cell ferroptosis through miR-34a-5p, indicating that exosomal miR-34a-5p may serve as a critical inducer of RIBE in B cells.

## Supplementary Material

Supplementary Material

Supplementary Material

Supplementary Material

Supplementary Material

Supplementary Material
