# Supplementary material for: B cell-derived exosomal miR-34a-5p mediates radiation-induced bystander effect through ferroptosis
Source: Open Med (Wars). 2026 Mar 20;21(1):20261375. doi: 10.1515/med-2026-1375 (PMC13007560; doi:10.1515/med-2026-1375)
Supplement: Supplementary file 1 — Supplementary Material [file j_med-2026-1375_suppl_001.docx]

**Table S1 Primer sequences.**

| **Primers** | **Sequence (5' to 3')** |
| --- | --- |
| U6-F | CGATACAGAGAAGATTAGCATGGC |
| U6-R | AACGCTTCACGAATTTGCGT |
| hsa-miR-138-5p-F | agctggtgttgtgaatcag |
| hsa-miR-138-5p-RT | GTCGTATCCAGTGCGTGTCGTGGAGTCGGCAATTGCACTGGATACGACCGGCCTG |
| hsa-miR-34a-5p-F | gcagtggcagtgtcttag |
| hsa-miR-34a-5p-RT | GTCGTATCCAGTGCGTGTCGTGGAGTCGGCAATTGCACTGGATACGACACAACCA |
| hsa-miR-625-3p-F | gcgcaggactatagaactttc |
| hsa-miR-625-3p-RT | GTCGTATCCAGTGCGTGTCGTGGAGTCGGCAATTGCACTGGATACGACTGAGGGG |
| hsa-miR-194-5p-F | cagtgtaacagcaactcca |
| hsa-miR-194-5p-RT | GTCGTATCCAGTGCGTGTCGTGGAGTCGGCAATTGCACTGGATACGACTCCACAT |
| hsa-miR-1183-F | cactgtaggtgatggtgaga |
| hsa-miR-1183-RT | GTCGTATCCAGTGCGTGTCGTGGAGTCGGCAATTGCACTGGATACGACTGCCCAC |
| SLC7A11-F | GGTCCATTACCAGCTTTTGTACG |
| SLC7A11-R | AATGTAGCGTCCAAATGCCAG |
| GPX4-F | GAGGCAAGACCGAAGTAAACTAC |
| GPX4-R | CCGAACTGGTTACACGGGAA |
| FTH1-F | AACCCGGCGCTCGTTCC |
| FTH1-R | AGTCCTGGTGGTAGTTCTGGC |
| CDKN1A-F | GAGACTCTCAGGGTCGAAAACG |
| CDKN1A-R | GACTAAGGCAGAAGATGTAGAGCG |
| GAPDH-F | ACAACTTTGGTATCGTGGAAGG |
| GAPDH-R | GCCATCACGCCACAGTTTC |
