# Supplementary material for: B cell-derived exosomal miR-34a-5p mediates radiation-induced bystander effect through ferroptosis
Source: Open Med (Wars). 2026 Mar 20;21(1):20261375. doi: 10.1515/med-2026-1375 (PMC13007560; doi:10.1515/med-2026-1375)
Supplement: Supplementary file 2 — Supplementary Material [file j_med-2026-1375_suppl_002.docx]

| AccID | log2FC | Pvalue | FDR | Style | IR-exo-1 | IR-exo-2 | IR-exo-3 | non-IR-exo-1 | non-IR-exo-2 | non-IR-exo-3 |
| --- | --- | --- | --- | --- | --- | --- | --- | --- | --- | --- |
| hsa-miR-20a-5p | 1.196883 | 1.16E-58 | 2.65E-56 | up | 29108 | 33744 | 25919 | 12069 | 10222 | 7567 |
| hsa-miR-33b-5p | 1.351545 | 1.22E-52 | 1.99E-50 | up | 2783 | 3291 | 2420 | 1003 | 982 | 594 |
| hsa-miR-138-5p | 1.335094 | 4.93E-48 | 6.26E-46 | up | 5081 | 6311 | 4819 | 1796 | 1989 | 1184 |
| hsa-miR-34a-5p | 1.196926 | 8.78E-41 | 6.28E-39 | up | 16045 | 17149 | 13392 | 5681 | 6400 | 3683 |
| hsa-miR-16-5p | 1.127996 | 3.59E-37 | 2.42E-35 | up | 21236 | 28783 | 22446 | 9937 | 9369 | 6323 |
| hsa-miR-590-3p | 1.619637 | 1.3E-31 | 6.77E-30 | up | 545 | 632 | 440 | 151 | 157 | 97 |
| hsa-miR-32-5p | 1.237682 | 2.38E-28 | 1.13E-26 | up | 928 | 1167 | 849 | 370 | 375 | 221 |
| hsa-miR-19a-3p | 1.316416 | 3.31E-24 | 1.15E-22 | up | 1454 | 1444 | 1072 | 412 | 543 | 279 |
| hsa-miR-9985 | 1.334394 | 1.75E-22 | 5.55E-21 | up | 641 | 597 | 502 | 209 | 181 | 142 |
| hsa-miR-625-3p | 1.145459 | 2.31E-21 | 6.61E-20 | up | 733 | 785 | 617 | 290 | 292 | 168 |
| hsa-miR-374a-3p | -1.82654 | 7.27E-89 | 8.32E-86 | down | 2197 | 2045 | 1680 | 6253 | 6130 | 3921 |
| hsa-let-7f-5p | -1.63654 | 1.01E-75 | 5.8E-73 | down | 563449 | 522565 | 405882 | 1415961 | 1267097 | 897428 |
| hsa-miR-148b-3p | -1.51552 | 7.29E-69 | 2.78E-66 | down | 99974 | 96869 | 73192 | 241784 | 205105 | 148878 |
| hsa-miR-151a-3p | -1.84455 | 4.83E-64 | 1.38E-61 | down | 15340 | 16641 | 12673 | 53743 | 35858 | 33344 |
| hsa-miR-148a-3p | -1.43484 | 4.17E-57 | 7.96E-55 | down | 4452448 | 4627541 | 3518929 | 10994736 | 8374760 | 6827603 |
| hsa-miR-106b-3p | -1.20149 | 2.88E-48 | 4.12E-46 | down | 38860 | 37888 | 29917 | 75475 | 65578 | 48169 |
| hsa-miR-532-5p | -1.02927 | 8.27E-48 | 9.46E-46 | down | 17288 | 18570 | 14203 | 30924 | 28848 | 19250 |
| hsa-miR-30a-3p | -1.38114 | 1.58E-47 | 1.64E-45 | down | 981 | 1120 | 905 | 2227 | 2412 | 1436 |
| hsa-miR-140-3p | -1.37332 | 5.81E-45 | 5.54E-43 | down | 54443 | 55690 | 41785 | 129501 | 94272 | 78892 |
| hsa-miR-1246 | -1.31516 | 2.24E-44 | 1.97E-42 | down | 227936 | 234286 | 180183 | 516968 | 386206 | 324959 |

**Table S2 The top 10 most upregulated and downregulated miRNAs between the non-IR-exo and IR-exo groups.**
