# Supplementary material for: B cell-derived exosomal miR-34a-5p mediates radiation-induced bystander effect through ferroptosis
Source: Open Med (Wars). 2026 Mar 20;21(1):20261375. doi: 10.1515/med-2026-1375 (PMC13007560; doi:10.1515/med-2026-1375)
Supplement: Supplementary file 3 — Supplementary Material [file j_med-2026-1375_suppl_003.docx]

**Supplemental files**

**Figure S1 Irradiated exosomal miR-34a-5p promotes ferroptosis of B cells.** (A-C) The effect of miR-34a-5p silencing in irradiated exosomes on the viability (A), LDH activity (B), and lipid peroxidation (C) of AHH-1 cells was measured using the CCK-8 assay, the LDH detection kit, and the BODIPY C-11 assay, respectively. All experiments were conducted with n=3, except for the CCK-8 assay, which was conducted with n=6. The data are depicted as the mean ± standard deviation (SD). ns, no significance. *, p <0.05; **, p <0.01.

**Figure S2 Schematic representation of the role and mechanism of irradiated exosomal miR-34a-5p in B cell ferroptosis.**
