# Supplementary figures and images for: B cell-derived exosomal miR-34a-5p mediates radiation-induced bystander effect through ferroptosis
Source: Open Med (Wars). 2026 Mar 20;21(1):20261375. doi: 10.1515/med-2026-1375 (PMC13007560; doi:10.1515/med-2026-1375)

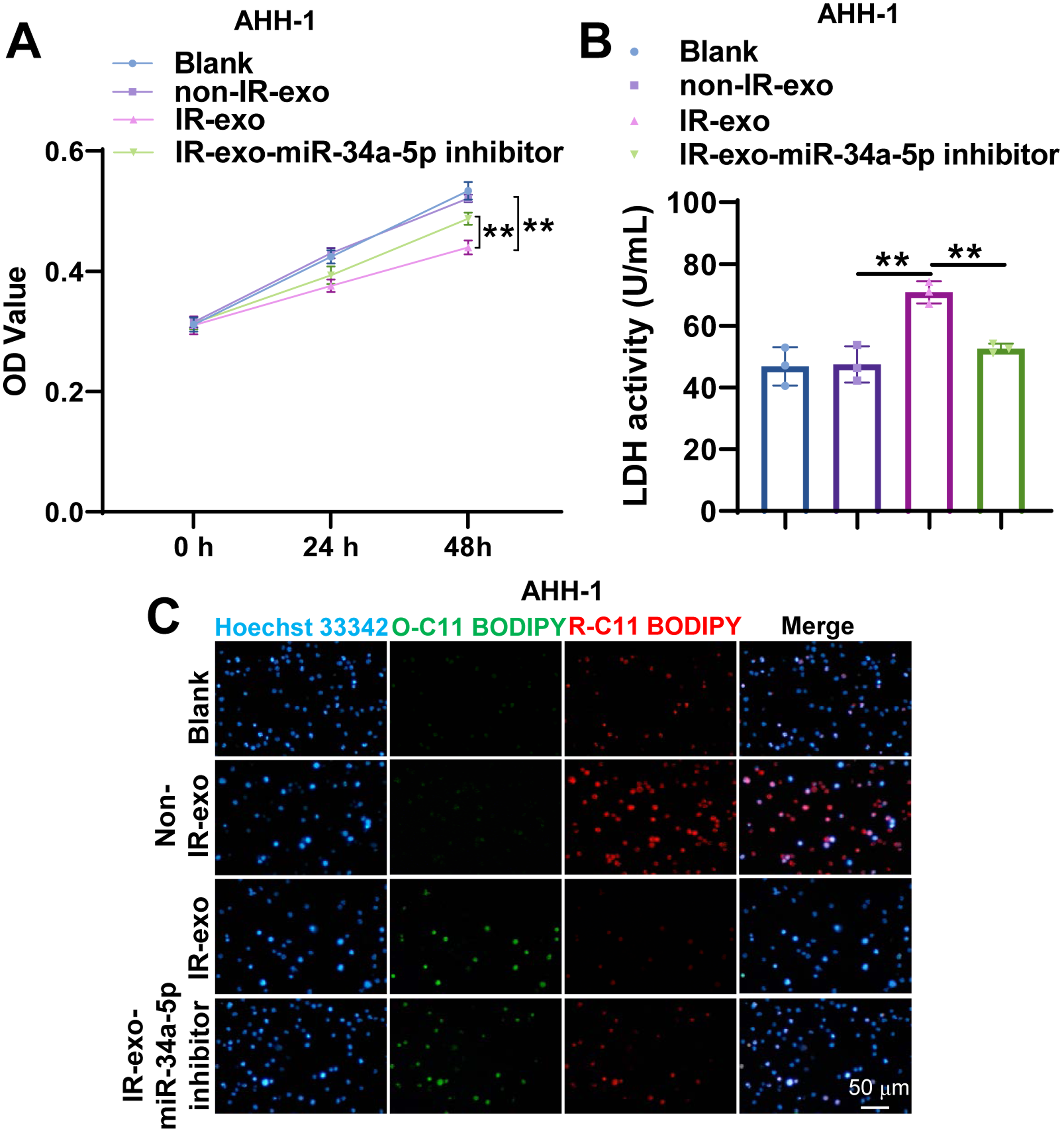

Supplement: Supplementary file 4 — Supplementary Material [file j_med-2026-1375_suppl_004.zip › j_med-2026-1375_suppl_004.tif]

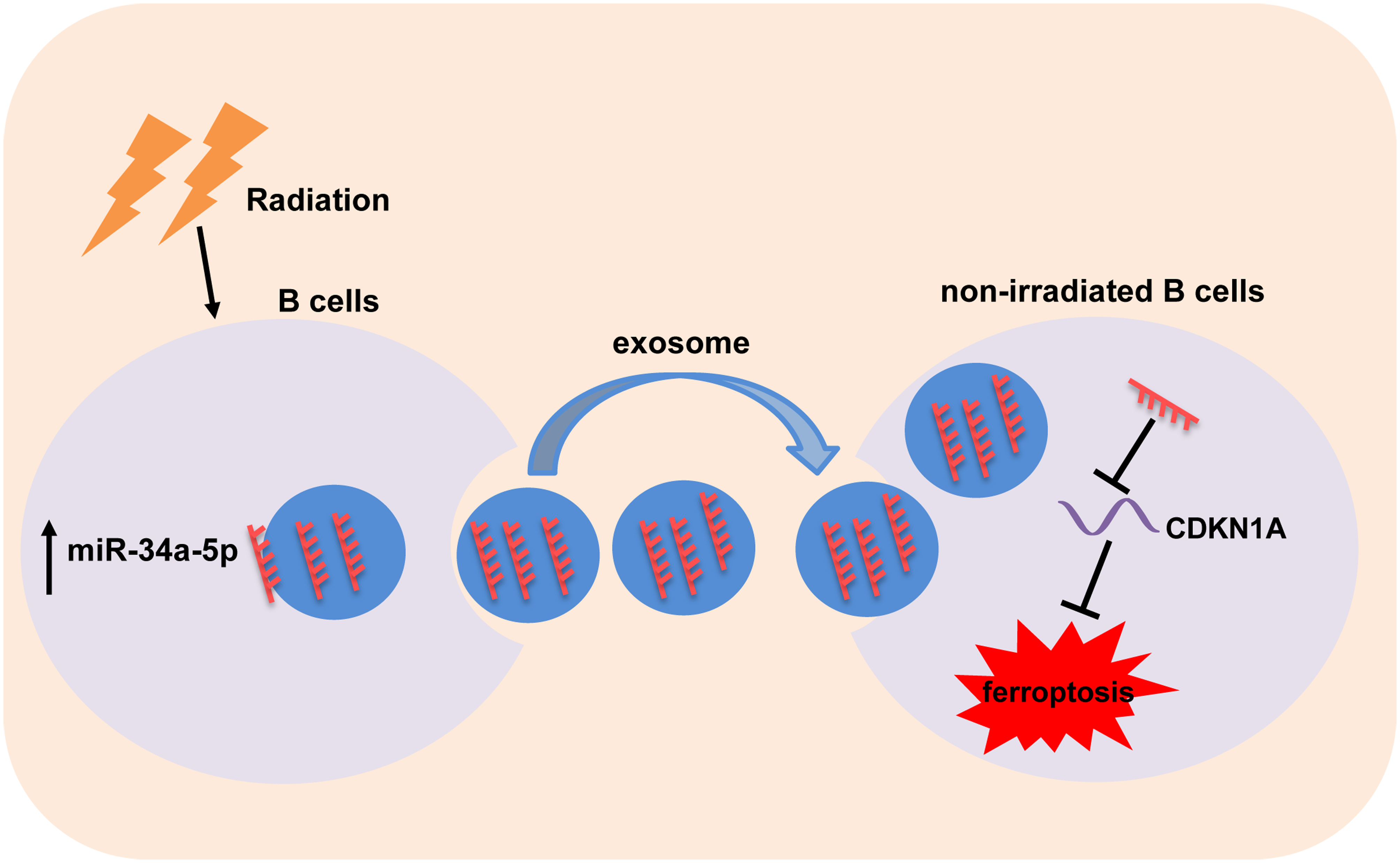

Supplement: Supplementary file 5 — Supplementary Material [file j_med-2026-1375_suppl_005.zip › j_med-2026-1375_suppl_005.tif]
